# Clinical Utility of the T2Candida Panel: A Real-World Analysis of More Than 2000 Cases

**DOI:** 10.1007/s11046-025-00979-x

**Published:** 2025-08-12

**Authors:** Felix Lötsch, Lukas Bouvier-Azula, Wolfgang Barousch, Iris Camp, Peter Starzengruber, Athanasios Makristathis, Birgit Willinger

**Affiliations:** 1 Division of Clinical Microbiology, Department of Laboratory Medicine, Medical University of Vienna, Vienna, Austria; 2 Comprehensive Center for Infection Medicine, Medical University of Vienna, Vienna, Austria; 3 Department of Hospital Epidemiology and Infection Control, Medical University of Vienna, Vienna, Austria

**Keywords:** T2Candida, T2 technology, Candida, Invasive candidiasis, Candidaemia, Yeast, PCR

## Abstract

**Objectives:**

Invasive candidiasis, including candidaemia, is associated with high morbidity and mortality. Diagnosis is traditionally based on blood culture, which lacks sensitivity. Therefore, additional tools such as PCR-based diagnostic methods are increasingly used. The T2MR technology is based on polymerase chain reaction and detection of the PCR product involving magnetic resonance technology. In this study, we compare the T2Candida in a clinical routine setting to conventional blood culture in order to explore its usefulness, strengths and weaknesses in its daily application.

**Methods:**

This retrospective analysis was performed at the Vienna University Hospital with clinical routine samples submitted between April 2021 and May 2024. Sensitivity, specificity, positive predictive value, negative predictive value and accordance were calculated with blood culture as reference method. Patients with a positive T2Candida result but a negative result in blood culture were assessed according to a clinical case definition. Based on direct detection in blood by alternative methods (e.g. blood culture, alternative PCR), 1-3-beta-D-Glucan, patient risk factors and detection of the same species in other sample materials, each result was categorised as “proven”, “probable”, “possible”, “improbable” or “not assessable”.

**Results:**

2105 samples from 1447 unique patients were submitted for analysis during the study period. 94 samples were positive (4.5%) in the T2Candida, with 4 samples positive for more than one target. 26 out of these 94 (27.7%) were also positive in blood culture. 339 (16.1%) samples were invalid. The most frequent species detected was *Candida albicans/tropicalis* with 57 detections. Overall sensitivity of the T2Candida panel in our setting was 0.62 (95% CI 0.41–0.80) and specificity was 0.96 (95% CI 0.95–0.97). Cases detected by the T2Candida panel were assessed as proven (n = 28), probable (n = 11), possible (n = 29), improbable (n = 15) and not assessable (n = 15). Median time-to-result was 3.9 h for the T2Candida compared to a median time-to-positivity of blood culture ranging from 22.7 to 42.0 h depending on the species.

**Conclusions:**

The introduction of the T2Candida panel led to a substantial rise in patients diagnosed with invasive candidiasis. Combination of both the T2Candida panel and conventional blood culture led to the detection of more positive samples than each test alone.

**Supplementary Information:**

The online version contains supplementary material available at 10.1007/s11046-025-00979-x.

## Introduction

Invasive candidiasis comprises both candidaemia and deep-seated candidiasis, and is associated with high morbidity and mortality [1]. For decades, blood culture has been the cornerstone of candidaemia diagnosis, enabling species identification and susceptibility testing. However, its utility is limited by low sensitivity and slow turnaround times, and it fails to diagnose deep-seated candidiasis in the absence of concurrent candidaemia. Also prior antifungal therapy can reduce the sensitivity of culture-based methods. To address these limitations, additional diagnostic tools such as serologic assays (e.g. 1-3-β-D-glucan (BDG) detection) and polymerase chain reaction (PCR)-based methods, are increasingly being explored and implemented in clinical practice [2]. These approaches promise to offer a more rapid and more sensitive diagnosis of invasive candidiasis, which is crucial given that early initiation of antifungal therapy has the potential to reduce mortality [3].

The T2 magnetic resonance method (T2MR) is a relatively new PCR-based diagnostic tool designed for the rapid detection of common *Candida* species directly from blood samples. It uses magnetic resonance (MR) technology to detect amplified DNA which binds to magnetic nanoparticles with complementary DNA probes. The fungal panel (T2Candida panel) detects *Candida albicans*/*tropicalis*, *C. parapsilosis* and *C. glabrata*/*krusei* (now renamed *Nakaseomyces glabratus* and *Pichia kudriavzevii*). The assay is designed to identify small amounts of target cells with a turnaround time of approximately three to five hours.

In the first multicentre clinical trial and a subsequent pooled analysis of all published data, the T2Candida panel demonstrated a sensitivity and specificity exceeding 90%, respectively [4, 5]. However, its diagnostic performance in real-world clinical settings, where its use is less restricted than in controlled research environments, remains largely unknown. Moreover, previous studies were limited by low sample sizes and/or very few cases of candidaemia. Between 2018 and 2020, we assessed the T2Candida panel in comparison to the Septifast assay and conventional blood culture in a prospective study in patients from intensive care units (ICU). The panel proofed to be a suitable alternative to the no longer available Septifast assay. Based on this data, it was later introduced as a diagnostic tool for the routine setting complementary to conventional blood culture. However, even in this research setting, the sensitivity was lower than in previous studies (63.6%; 95% CI 41–83%) [6]. In this analysis now, we evaluate the performance of the T2Candida panel applied in a clinical routine setting at a large tertiary care centre in Vienna, Austria. We compare it to conventional blood culture in order to explore its clinical utility, strengths and limitations in daily practice.

## Methods

This retrospective analysis explores the performance of the T2Candida Panel in comparison to conventional blood culture at Vienna University Hospital (AKH Wien). All patients, regardless of age, ward, or diagnosis, who underwent T2Candida testing between April 2021 and May 2024 were included. The Vienna University Hospital is the largest hospital in Austria and serves as an academic reference centre providing tertiary care to Eastern Austria. Consequently, the population in this analysis includes all major risk groups for invasive fungal infections, including transplant recipients, patients with hematologic malignancies, and individuals undergoing abdominal surgery.

Since April 2021, the T2Candida Panel has been available to clinicians without restrictions on patient selection or testing frequency, with indications determined solely by the attending physicians. Sensitivity, specificity, positive predictive value (PPV), negative predictive value (NPV) and accordance with blood culture were calculated for all available T2Candida Panel results (including multiple tests per patient) in comparison to a single pair of blood cultures obtained within ±1 day of the T2Candida sample’s arrival at the laboratory. If multiple blood culture pairs were submitted within this timeframe, only the first pair was used for comparison. The analysis was repeated using any corresponding blood culture obtained within ±3 days of the T2Candida sample's arrival. For this analysis, only the first T2Candida result per patient was included. Additionally, Kaplan–Meier curves and survival analysis were performed to compare patients with a positive T2Candida result to those with a positive blood culture where no T2Candida test had been requested at all.

Whole blood samples for the T2Candida Panel were collected in 4 mL VACUETTE® K3 EDTA tubes (Greiner Bio-One, Kremsmünster, Austria). Until June 2023, samples were processed Monday to Friday from 8:00 a.m. to 7:00 p.m. and on weekends and holidays from 8:00 a.m. to 3:00 p.m. Samples arriving outside these hours were stored at 2–8 °C and processed the following day. From July 2023 onward, testing was performed continuously, 24/7. All analyses were conducted using two onsite T2Dx® devices (T2 Biosystems, Lexington, MA, USA). Blood cultures were collected in aerobic and anaerobic BACT/ALERT® FA Plus bottles (bioMérieux, Marcy-l’Étoile, France) and incubated in a BacT/ALERT 3D system (bioMérieux, Marcy-l’Étoile, France) for up to seven days. Upon detection of a positive signal, blood culture bottles were processed according to the standard operating procedures of the Division of Clinical Microbiology, Medical University of Vienna. Species identification was performed using matrix-assisted laser desorption/ionization time-of-flight (MALDI-TOF) mass spectrometry on a MALDI Biotyper® Sirius system (Bruker Daltonik GmbH, Bremen, Germany).

Data on T2Candida panel results and corresponding blood cultures were extracted from the laboratory information system (MOLIS). Patient data were retrieved from the hospital’s electronic medical records system (AKIM). Patients with a positive T2Candida result were classified according to their probability of having true invasive candidiasis based on the case definition outlined in Table 1. All statistical analyses were performed using R (Version 4.2.2) with the following packages: “readxl”, “dplyr”, “ggplot2”, “tidyr,” “HH”, “lubridate”, “survival”, “survminer” and “purrr.” The study received ethical approval from the Ethics Committee of the Medical University of Vienna (EK: 1802/2024).

**Table 1:** Case definitions for patients with a positive T2Candida result

Category	Definition
Proven	Positive blood culture (± 7d) with identical species as T2Candida **OR** Positive central venous catheter tip (± 7) with identical species as T2Candida **OR** Positive alternative PCR from blood with identical species as T2Candida
Probable	Positive BDG (± 7d) **AND** detection of the same *Candida* sp. as T2Candida from another body site (± 7d) (excluding superficial swabs) **OR** Positive BDG (± 7d) **AND** risk factors (hematologic malignancies with and without neutropenia, neutropenia following HSCT, and certain patients in the ICU who are at higher risk (> 10%) for IC as a result of gastrointestinal surgery with recurrent anastomotic leaks, perforations of the upper gastrointestinal tract, or necrotizing pancreatitis)
Possible	Positive BDG (± 7d) **OR** underlying risk factor (hematologic malignancies with and without neutropenia, neutropenia following HSCT, and certain patients in the ICU who are at higher risk (> 10%) for IC as a result of gastrointestinal surgery with recurrent anastomotic leaks, perforations of the upper gastrointestinal tract, or necrotizing pancreatitis) **OR** isolation of the same *Candida* sp. as T2Candida from another body site WITHOUT fulfilling criteria for any other category
Improbable	At least 2 out of 3 of the following criteria Negative BDG No isolation from another body site No risk factor
Not assessable	Insufficient data for classification into another category

BDG = 1-3-beta-D-Glucan; HSCT = haematopoietic stem cell transplantation; ICU = intensive care unit; IC = invasive candidiasis

For consistency and readability, we have used the previous species nomenclature throughout this manuscript (i.e., *Candida glabrata* instead of *Nakaseomyces glabratus* and *Candida krusei* instead of *Pichia kudriavzevii*). Language editing assistance was provided by ChatGPT (GPT-4-turbo, OpenAI).

## Results

### Routine Use

**Fig. 1:**
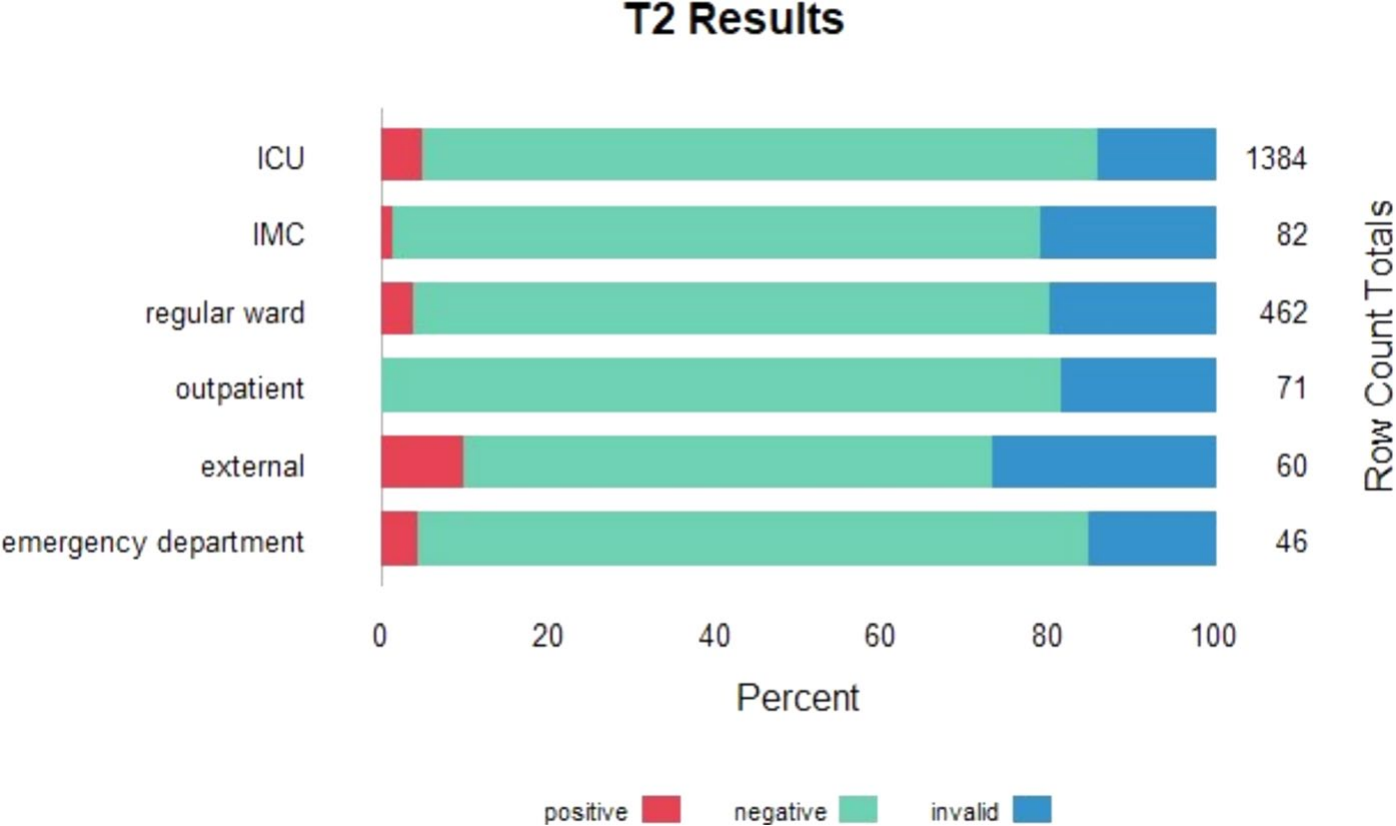
Number of positive, negative and invalid T2Candida results per type of submitting ward

**Fig. 2:**
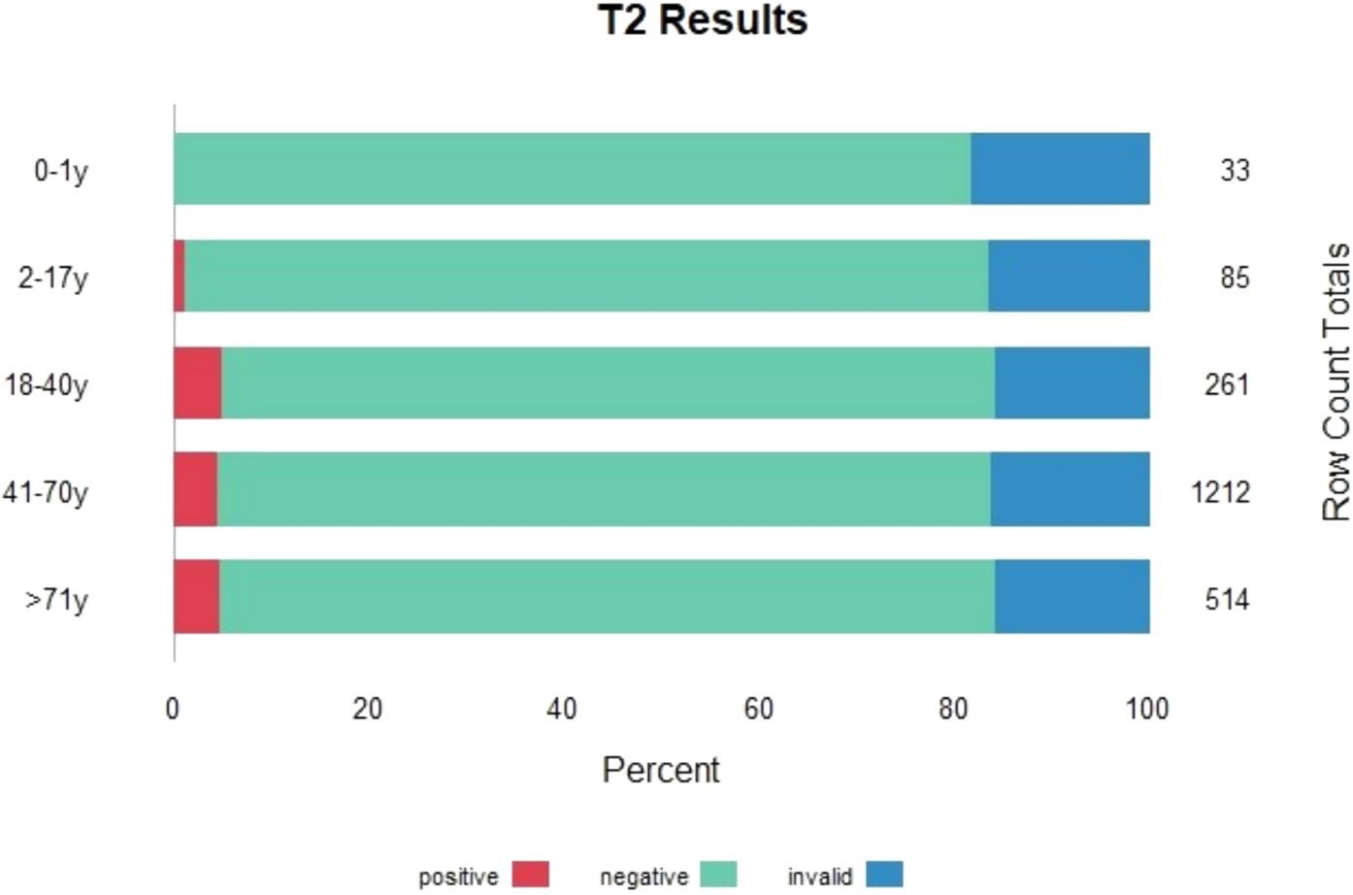
Number of positive, negative and invalid T2Candida results per age group

Between April 2021 and May 2024, the T2Candida panel was performed 2105 times on blood samples from 1447 unique patients. The majority of samples were submitted by ICUs (n = 1384). The number of tests per patient is presented in Supplementary Table S1. In total, 94 samples (4.5%) yielded a positive result, indicating that approximately 22 patients need to be tested to obtain one positive T2Candida result. In four of these 94 positive samples (4%), two different *Candida* species were identified, resulting in a total of 98 detected targets. *Candida albicans*/*C. tropicalis* was detected 57 times, *C. parapsilosis* 29 times, and *C. krusei*/*C. glabrata* 12 times. A total of 339 samples (16.1%) were classified as invalid, while 1672 (79.4%) were negative. The proportion of invalid results remained high but stable over time, with proportions of 15.7% in 2021, 12.2% in 2022, 19.6% in 2023, and 18.1% in 2024. Positive results were uncommon, particularly in younger patients, while the proportion of invalid results remained consistently high, regardless of the submitting ward or patient age (see Figs. 1, 2).

### Diagnostic Performance Compared to Blood Culture

We compared all T2Candida results to a single pair of blood cultures collected concurrently. The closest available pair of blood cultures within a maximum of ±1 day before or after the respective T2Candida test was included. Overall results are presented in Table 2, while target-specific results are detailed in Supplementary Tables S2–S4. For this analysis, the sample size was lower, as only T2Candida results with a corresponding blood culture were included (n = 1818). According to Table 2, 28.4 patients need to be tested to identify one additional positive result that would not have been detected by blood culture.

**Table 2:** Diagnostic performance of the T2Candida panel with the single closest pair of blood cultures ±1 day as reference

Combined species	Single pair of blood culture		
	Positive	Negative	Total
T2 (all episodes)			
Positive	16	64	80
Negative/invalid	10	1728	1738
Total	26	1792	1818
Sensitivity	0.62 (95% CI 0.41–0.80)		
Specificity	0.96 (95% CI 0.95–0.97)		
PPV	0.20 (95% CI 0.12–0.30)		
NPV	0.99 (95% CI 0.99–1.00)		
Agreement	0.96 (95% CI 0.95–0.97)		

Subsequently as a sensitivity analysis, the first T2Candida result for each patient was compared to the results of any blood culture performed within ±3 days of the T2Candida testing date. Blood cultures were interpreted as positive if at least one bottle showed growth. The results are presented in Table 3. The sample size in this analysis is lower as only the first T2Candida result of each patient was included.

**Table 3:** Diagnostic performance of the first T2Candida testing per patient with any pair of blood cultures within ±3 days as reference

Combined species	Any pair of blood culture ± 3 days		
	Positive	Negative	Total
T2 (first episodes)			
Positive	12	38	50
Negative/invalid	10	1267	1277
Total	22	1305	1327
Sensitivity	0.55 (95% CI 0.32–0.76)		
Specificity	0.97 (95% CI 0.96–0.98)		
PPV	0.24 (95% CI 0.13–0.38)		
NPV	0.99 (95% CI 0.99–1.00)		
Agreement	0.96 (95% CI 0.95–0.97)		

The lower number of positive blood cultures compared to Table 2 is explained by the fact that only the first T2Candida of any patient was included leading to a lower overall sample size

We then individually assessed patients with a positive T2Candida result to determine the likelihood of true invasive candidiasis based on a clinical case definition (see “Methods”). The results are presented in Table 4.

**Table 4:** Case categorization of each positive T2Candida result per species

Target	Proven	Probable	Possible	Improbable	Not assessable	Total
*C. albicans/C. tropicalis*	23	9	16	2	7	57
*C. parapsilosis*	2	1	11	10	5	29
*C. glabrata/C. krusei*	3	1	2	3	3	12

Among all assessable patients, only 2 out of 50 (4%) samples positive for *C. albicans/C. tropicalis* were judged as unlikely to represent true invasive candidiasis, compared to 10 out of 24 (42%) for *C. parapsilosis* and 3 out of 9 (33%) for *C. glabrata/C. krusei*. A comparison of the diagnostic yield, including only T2Candida results classified as proven, probable, or possible invasive candidiasis, alongside blood culture results, is presented in Table 5 and Fig. 5.

**Table 5:** Diagnostic yield of the T2Candida compared to blood culture

	T2 (proven, probable or possible)	T2 (proven or probable)	Single pair of blood culture	Ratio A (proven, probably and possible vs. blood culture)	Ratio B (proven and probable vs. blood culture)
*C. albicans/C. tropicalis*	48/1818 (2.6%)	32/1818 (1.8%)	19/1818 (1.0%)	2.5 (95% CI 1.49–4.30)	1.7 (95% CI 0.95–2.97)
*C. parapsilosis*	14/1818 (0.8%)	3/1818 (0.2%)	2/1818 (0.1%)	7.0 (95% CI 1.59–30.80)	1.5 (95% CI 0.25–8.98))
*C. glabrata/C. krusei*	6/1818 (0.3%)	4/1818 (0.2%)	5/1818 (0.3%)	1.2 (95% CI 0.37–3.93)	0.8 (95% CI 0.21–2.98)

**Fig. 3:**
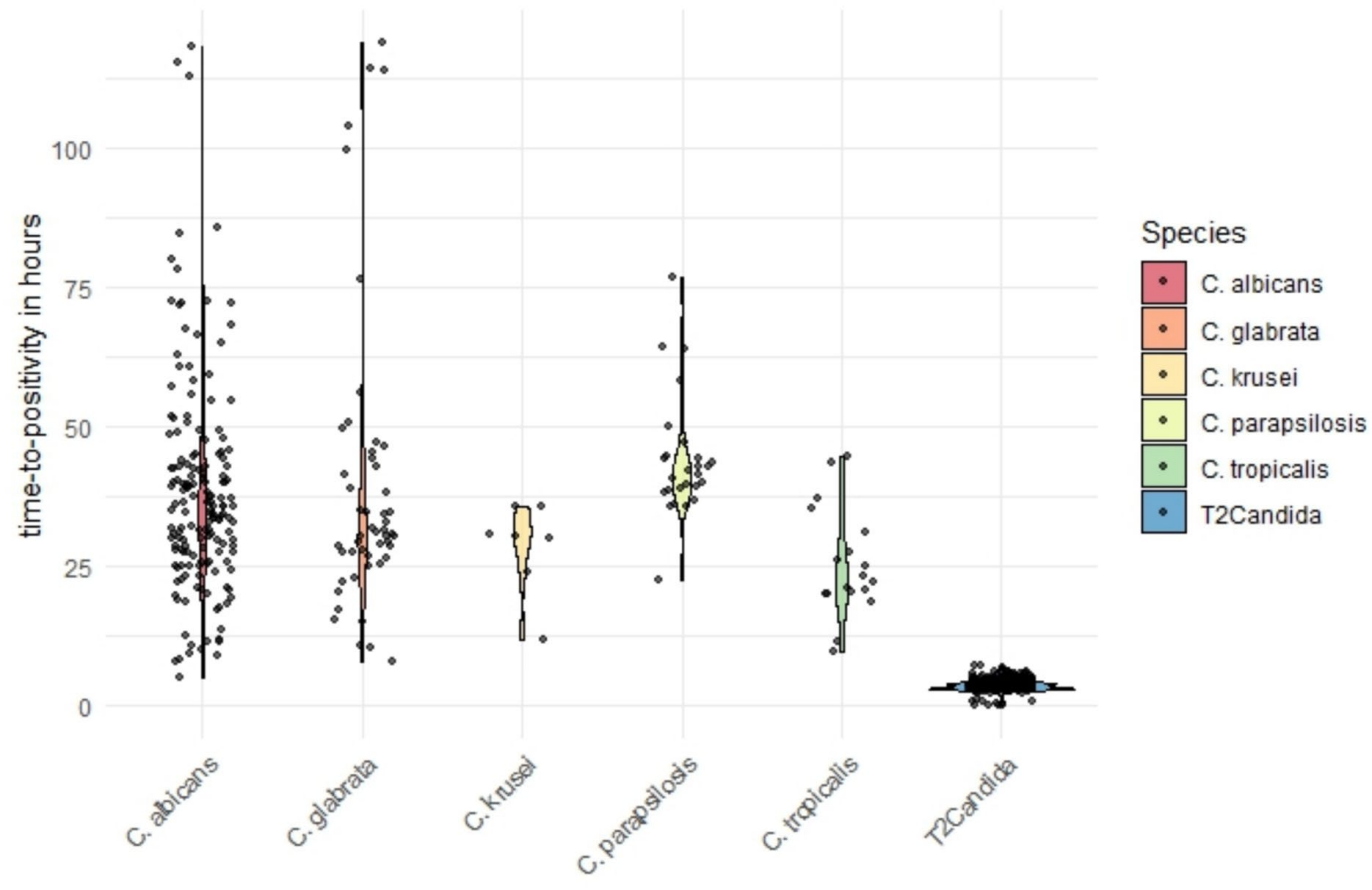
Time-to-positivity in blood culture for different Candida species and Time-to-result of T2Candida

The median time to positivity for blood cultures was 35.4 h for *C. albicans*, 22.7 h for *C. tropicalis*, 42.0 h for *C. parapsilosis*, 29.3 h for *C. glabrata* and 30.6 h for *C. krusei*. In contrast, the median time to result for the T2Candida was 3.9 h (see Fig. 3).

**Fig. 4:**
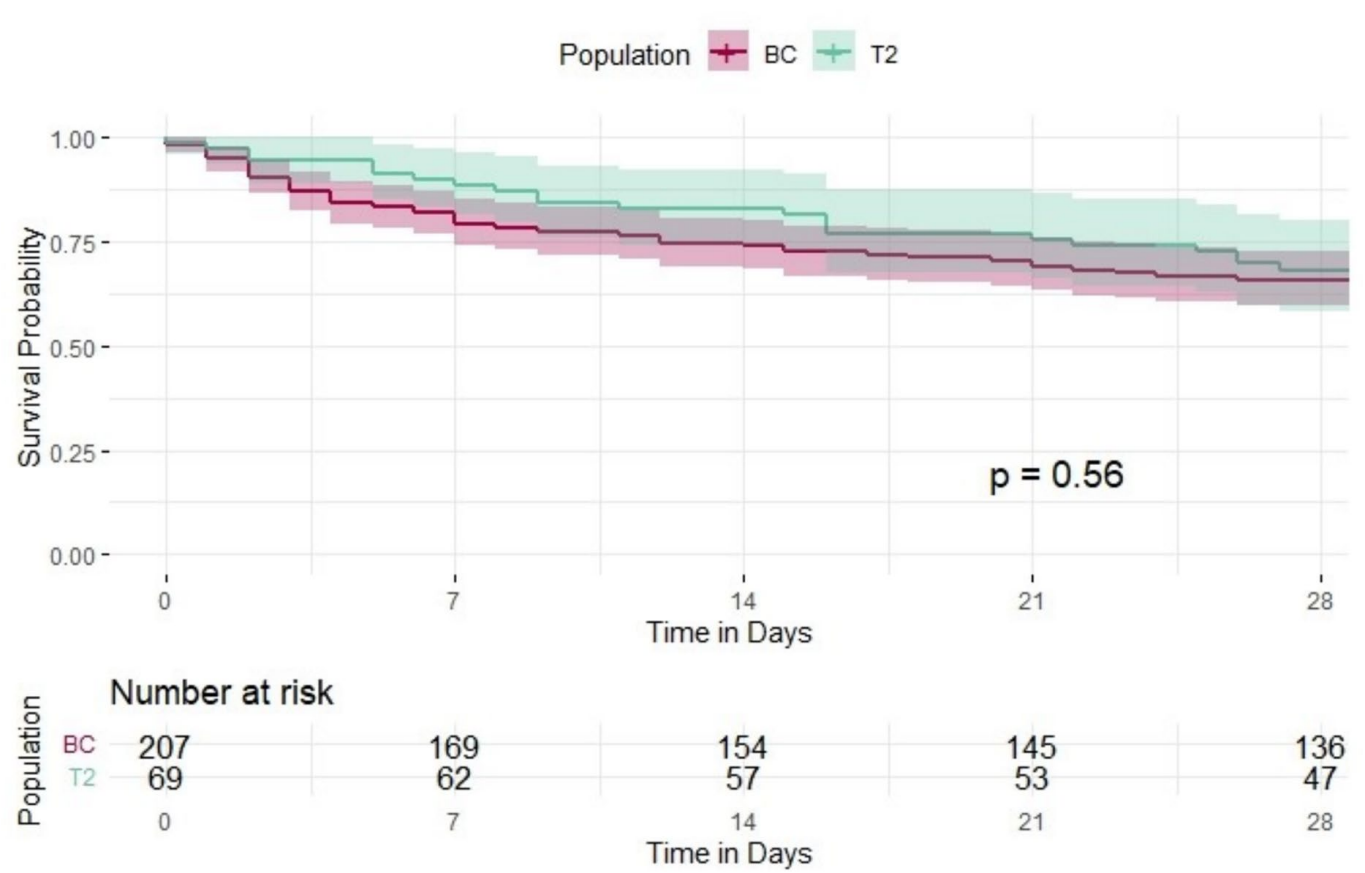
Kaplan–Meier plot showing the survival of patients with a positive T2Candida (green) compared to patients with positive blood culture, where no T2Candida was performed (red)

The median time to positivity for blood cultures with a corresponding negative T2Candida was 34.0 h, compared to 33.6 h for blood cultures with a corresponding positive T2Candida test result. We then compared the 28-day mortality between patients with a positive T2Candida result and those with a positive blood culture of a target species where no T2Candida had been performed at all. Only the first T2Candida or blood culture result of each patient was considered as day 0. A total of 69 individual patients treated at our institution had a positive T2Candida result, while 207 patients had a positive blood culture without any corresponding T2Candida testing. The overall survival at day 28 in the T2Candida group was 68% compared to 65% in the blood culture only group. There was no significant difference in 28-day survival, as shown in the Kaplan Meier plot (*p* = 0.56) (see Fig. 4). In a Cox regression model adjusted for age and ward, the diagnostic method was not associated with a survival benefit (HR 0.72; 95% CI 0.43–1.19; *p* = 0.20).

## Discussion

**Fig. 5:**
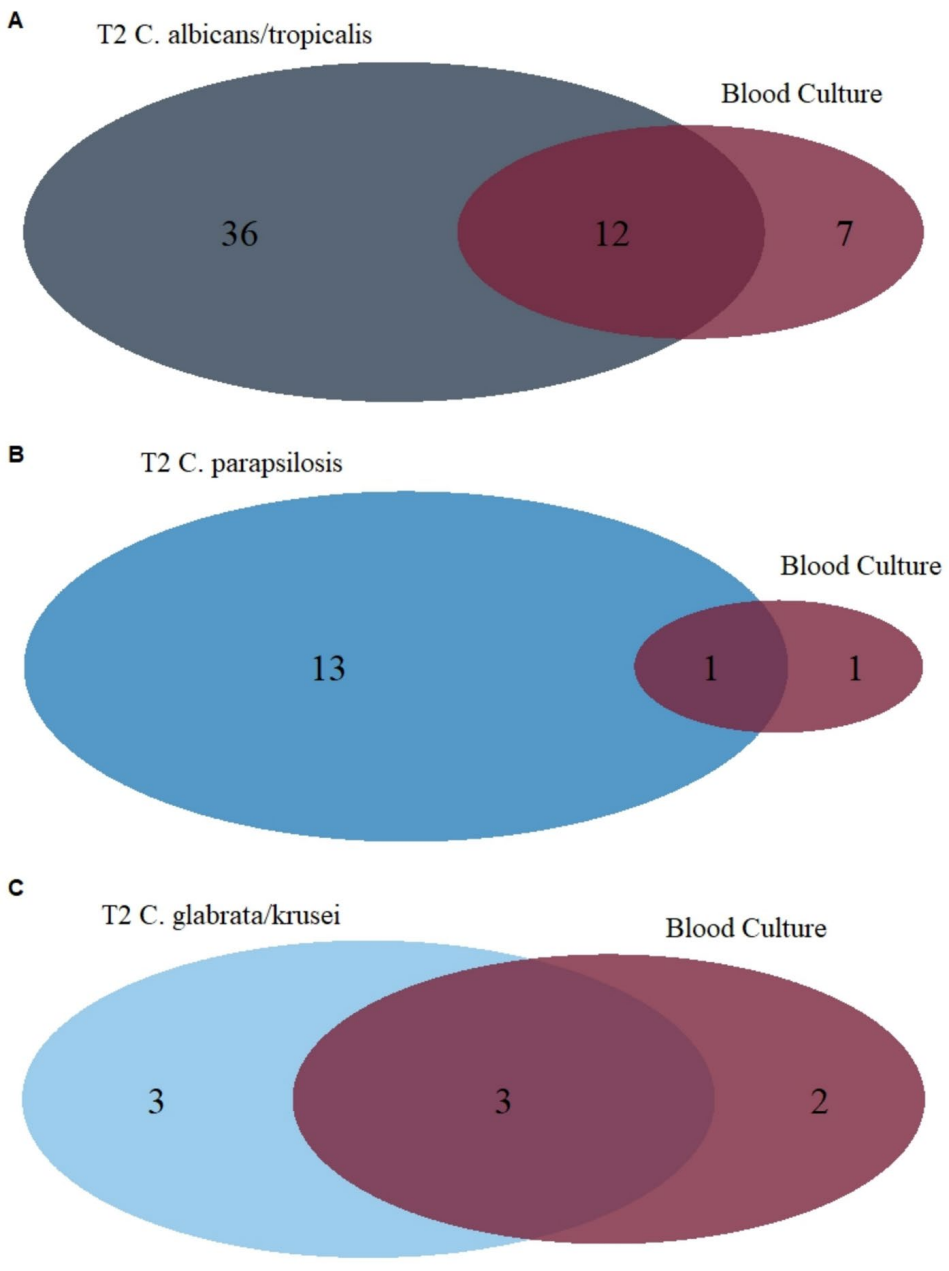
Venn plots showing cases detected by the T2, both methods and blood culture only. Only proven, probably and possible cases are shown

In this analysis we assess the T2Candida in a real-world setting in the largest cohort so far, which is often fundamentally different compared to a study setting. For example, there is no control over the amount of blood that is filled into blood culture bottles or the site of sampling. In this study, we demonstrated that supplementing conventional blood culture diagnostics with the T2Candida significantly increased the detection of *Candida* species in the bloodstream, especially of *C. albicans*. Among patients where both the T2Candida and blood cultures were sampled (see Table 2), 80 patients had a positive T2Candida result compared to only 26 patients with a positive blood culture. However, the overall sensitivity of the T2Candida compared to a single pair of blood cultures was only 0.62 (95% CI 0.41–0.80) in this real-world setting, which is markedly lower than the sensitivity reported in a previous meta-analysis (0.91; 95% CI 0.88–0.94) [4]. The findings of this meta-analysis were largely influenced by the single largest study on the T2Candida panel, in which some samples were artificially spiked due to the low number of actual candidaemia cases [5]. To date, only one study has reported an even lower sensitivity of 39% (95% CI 0.22–0.59) [7]. However, our calculated sensitivity is nearly identical to that reported previously from our centre, where the T2Candida was evaluated against blood culture in a study setting including patients from ICUs with a much higher proportion of positive samples (21.1%) [6]. In that study, the sensitivity of the T2Candida in confirmed cases of candidaemia was 63.6% (95% CI 41–83%). Additionally, a study from Denmark involving ICU patients at high risk for invasive candidiasis found that the combination of the T2Candida and blood culture was superior to either test alone [8], further supporting our findings and emphasizing the benefit of integrating both diagnostic methods (Fig. 5).

It remains unclear why the overall sensitivity of the T2Candida test is suboptimal despite detecting more cases of candidaemia than conventional blood cultures. Differences in sampling dates and time points are unlikely to be a major factor, as we applied a narrow reference window of ±1 day for blood cultures. Unfortunately, we were unable to assess whether blood for the T2Candida Panel was drawn from a different site than the corresponding blood culture bottles (e.g., peripheral vs. central sampling), which could have influenced the results. These findings suggest that the enrichment of pathogens through incubation in blood culture bottles remains crucial in certain cases. Additionally, variations in blood culture sensitivity across laboratories may contribute to the discrepancies in reported sensitivities between different studies. A higher sensitivity of blood cultures in certain settings could lead to a comparatively lower sensitivity of the T2Candida test. Consequently, due to the unreliability of negative results and the limited scope of the panel, the T2Candida is unsuitable as a stand-alone test, including for antimicrobial stewardship aimed at reducing unnecessary antifungal therapy. A previous study investigating the impact of the T2Candida on antifungal prescribing found that empirical therapy was discontinued in only 33% of patients following a negative T2 result [9]. In light of our findings, a negative T2 result alone appears insufficient to justify stopping empirical therapy. However, its potential role in combination with other diagnostic tests (e.g., BDG and/or blood culture) warrants further investigation.

In our study population, the overall diagnostic yield was low, with only 4.5% of all samples testing positive. This suggests a relatively liberal use of the T2Candida at our institution. For example, its use in outpatients (excluding the emergency department) proved ineffective, as no positive samples were detected since the test’s introduction. Unfortunately, due to limited patient-level data, we were unable to identify subgroups with a higher proportion of positive results. To improve efficiency, future efforts should focus on defining patient populations with a higher pre-test probability.

When assessing the likelihood that T2Candida-positive but blood culture-negative results were true positives we observed species-specific differences. While most cases of *C. albicans*/*C. tropicalis* were considered as at least possible cases, many *C. parapsilosis* results appeared implausible. Nearly all of these patients were ICU patients (data not shown), and we speculate that blood was often drawn from central venous lines. Given that *C. parapsilosis* is a common skin colonizer, contamination—potentially via the connection hub—may explain these findings [10]. A possible explanation for the substantial higher number of positive results by the T2Candida are deep-seated infections without concurrent candidaemia. If DNA is shed into the bloodstream, the infection may be picked up by the molecular panel whereas blood culture requires viable *Candida* cells in the bloodstream. As deep-seated infections are true infections requiring therapy, we consider this as an additional benefit of the test.

We also observed a strikingly high number of invalid results. In the first pivotal study evaluating the T2Candida (8), 245 out of 2264 samples (10.8%) were invalid, compared to 16.1% in our study. Invalid results remained consistently high throughout the study period, regardless of the submitting ward or patient age. This makes patient-related factors or interactions with certain substances (e.g., drugs, parenteral nutrition) less likely as the primary cause. Although we were unable to retrieve device-specific data explaining the invalid results, Mylonakis et al. identified invalid internal controls and instrument malfunctions as the main reasons [5]. An internal quality control audit at our institution examined potential user-dependent factors—including iron supplementation, MRI contrast agents, parenteral nutrition, insufficient tube filling, and incorrect test tube use—but found no clear association.

A major advantage of the T2Candida over blood cultures is its significantly shorter turnaround time. While the median time-to-positivity for blood cultures exceeded 20 h for all species, the T2Candida provided results in a median of just 3.9 h (3 h 54 min). This faster detection could facilitate earlier initiation of targeted antifungal therapy. To assess this potential benefit, we compared patients with a positive T2Candida result to those with a positive blood culture where no T2Candida test was performed at all. Although mortality was slightly lower in the T2Candida group, the difference was not statistically significant (*p* = 0.56). This finding remained consistent after adjusting for age and ward. As data on empirical antifungal therapy were not available in our analysis, we cannot assess the impact of the test. In a setting where antifungal treatment is initiated at a low threshold, faster *Candida* sp. detection alone may not necessarily lead to earlier treatment or improved outcomes. In our institution, positive results were communicated immediately to the treating physician. However, the T2 result was not linked to an antimicrobial stewardship (AMS) intervention but left for interpretation by the treating physicians. For different rapid tests, linked AMS interventions were shown to be essential for a clinical impact [11].

A major challenge in analysing clinical data on diagnostic tests for invasive candidiasis, including this study, is the lack of universally applicable clinical case definitions that encompass diverse patient populations (e.g., those with neutropenia, recent abdominal surgery, or prolonged ICU stays). Where available, existing case definitions often rely on positive blood cultures. For example, an international consensus defines proven candidaemia in ICU patients as a positive blood culture from venepuncture [12]. Consequently, the T2Candida and blood cultures can only be compared against each other, rather than against an independent case definition that excludes these methods. To assess discrepant cases, we developed our own case definition based on international criteria [12, 13]. However, a key limitation is that this definition has not been externally validated. Additionally, as this study was laboratory-based, we lacked detailed patient data for subgroup analyses. Despite these limitations, our study is the first to evaluate the utility and performance of the T2Candida in a large real-world cohort with unrestricted test use. Institutions that apply the test selectively in high-risk subgroups may have different findings.

In summary, the introduction of the T2Candida at our institution significantly increased the number of diagnosed candidaemia cases. The combination of the T2Candida and conventional blood cultures detected more positive cases than either method alone. In our study, there was a non-significant trend toward improved 28-day survival in patients tested with the T2Candida compared to those diagnosed via blood culture alone. However, the potential impact of empirical antifungal therapy could not be assessed. Future research should explore the role of this technology as a tool for antimicrobial stewardship in combination with other methods.

## Supplementary Information

Below is the link to the electronic supplementary material.

Supplementary file1 (DOCX 14 kb)
