# Supplementary material for: Clinical Utility of the T2Candida Panel: A Real-World Analysis of More Than 2000 Cases
Source: Mycopathologia. 2025 Aug 12;190(5):71. doi: 10.1007/s11046-025-00979-x (PMC12343663; doi:10.1007/s11046-025-00979-x)
Supplement: Supplementary file 1 — Supplementary file1 (DOCX 14 kb) [file 11046_2025_979_MOESM1_ESM.docx]

# Supplementary Tables and Graphs

| Number of T2 panels per patient | n |
| --- | --- |
| 1 | 1092 |
| 2 | 205 |
| 3 | 71 |
| 4 | 41 |
| 5 | 22 |
| 6 | 6 |
| 7 | 4 |
| 8 | 2 |
| 9 | 4 |

**Supplementary Table 1:** Number of T2 Candida panels per patient

| ***C. albicans / tropicalis*** | | **Single pair of blood culture** | |  |
| --- | --- | --- | --- | --- |
|  |  | **Positive** | **Negative** | **Total** |
| **T2** | **Positive** | 12 | 38 | **50** |
|  | **Negative / Invalid** | 7 | 1761 | **1768** |
|  | **Total** | **19** | **1799** | **1818** |
|  | **Sensitivity** | 0.63 (95% CI 0.38-0.84) |  |  |
|  | **Specificity** | 0.98 (95% CI 0.97-0.99) |  |  |
|  | **PPV** | 0.24 (95% CI 0.13 – 0.38) |  |  |
|  | **NPV** | 1.00 (95%CI 0.99 - 1.00) |  |  |
|  | **Agreement** | 0.98 (95% 0.97 - 0.98) |  |  |

**Supplementary Table 2:** Diagnostic performance of the T2Candida Panel restricted to C. albicans / tropicalis.

| ***C. parapsilosis*** | | **Single pair of blood culture** | |  |
| --- | --- | --- | --- | --- |
|  |  | **Positive** | **Negative** | **Total** |
| **T2** | **Positive** | 1 | 24 | **25** |
|  | **Negative / Invalid** | 1 | 1792 | **1793** |
|  | **Total** | **2** | **1816** | **1818** |
|  | **Sensitivity** | 0.50 (95% CI 0.01-0.99) |  |  |
|  | **Specificity** | 0.99 (95% CI 0.98-0.99) |  |  |
|  | **PPV** | 0.04 (95% CI 0.00 – 0.20) |  |  |
|  | **NPV** | 1.00 (95%CI 1.00 - 1.00) |  |  |
|  | **Agreement** | 0.99 (95% 0.98 - 0.99) |  |  |

**Supplementary Table 3:** Diagnostic performance of the T2Candida Panel restricted to C. parapsilosis

| ***C. glabrata / krusei*** | | **Single pair of blood culture** | |  |
| --- | --- | --- | --- | --- |
|  |  | **Positive** | **Negative** | **Total** |
| **T2** | **Positive** | 3 | 6 | **9** |
|  | **Negative / Invalid** | 2 | 1807 | **1809** |
|  | **Total** | **5** | **1813** | **1818** |
|  | **Sensitivity** | 0.60 (95% CI 0.15-0.95) |  |  |
|  | **Specificity** | 1.00 (95% CI 0.99-1.00) |  |  |
|  | **PPV** | 0.33 (95% CI 0.07 – 0.70) |  |  |
|  | **NPV** | 1.00 (95%CI 1.00 - 1.00) |  |  |
|  | **Agreement** | 1.00 (95% 0.99 – 1.00) |  |  |

**Supplementary Table 4:** Diagnostic performance of the T2Candida Panel restricted to C. glabrata / krusei.
